# Platelet-rich plasma affects the proliferation of canine bone marrow-derived mesenchymal stromal cells in vitro

**DOI:** 10.1186/s12917-019-2010-x

**Published:** 2019-07-30

**Authors:** Sony Pandey, Dawn U. Hickey, Marti Drum, Darryl L. Millis, Maria Cekanova

**Affiliations:** 1 Department of Small Animal Clinical Siences, College of Veterinary Medicine, The University of Tennessee, Knoxville, 37996 TN USA; 2 UT-ORNL Graduate School of Genome Science and Technology, The University of Tennessee, Knoxville, 37996 TN USA

**Keywords:** Canine, Cell viability, Platelet-rich plasma, Osteoarthritis, Regenerative medicine, Multipotent mesenchymal stromal cells, Bone marrow

## Abstract

**Background:**

Reported efficacy of platelet-rich plasma (PRP) in regenerative medicine is contradictory. We validated the effects of PRP on proliferation of canine bone marrow-derived multipotent mesenchymal stromal cells (K9BMMSCs) in vitro. PRP was extracted from blood of six dogs with osteoarthritis. K9BMMSCs were established from bone marrow and characterized for CD90 and CD19 expression by immunocytochemistry. Effects of PRP concentrations on viability of matching autologous K9BMMSCs were validated using MTS assay.

**Results:**

Positive CD90 and negative CD19 expression confirmed MSC origin. PRP at 40% volume/volume concentration increased, while PRP at 80 and 100% v/v concentrations suppressed viability of tested K9BMMSCs.

**Conclusion:**

PRP concentration plays an important role in K9BMMSCs viability, which could affect tissue repairs in vivo.

**Electronic supplementary material:**

The online version of this article (10.1186/s12917-019-2010-x) contains supplementary material, which is available to authorized users.

## Background

Platelet-rich plasma (PRP) is an enriched plasma containing variety of growth factors, including the platelet derived growth factor (PDGF), vascular endothelial growth factor (VEGF), transforming growth factor-β (TGF-β), fibroblast growth factor (FGF), and insulin-like growth factors I and II (IGF-I, IGF-II) [1, 2]. These growth factors are potent chemoattractant and mitogens, which help attract and activate surrounding cells at sites of injury. Importantly, at sites of injury, PRP entraps mesenchymal cells and supports the proliferation and differentiation of surrounding endothelial, and other stromal cells resulting in accelerated wound healing [1, 3–7]. The proliferation and differentiation potentials of the multipotent mesenchymal stromal cells (MSCs) can be applied for the treatment of degenerative diseases, including osteoarthritis (OA) [8].

OA is a painful and debilitating orthopedic condition, affecting both humans [9] and companion animals [10–12]. This chronic disease is most commonly treated by anti-inflammatory drugs, pain relievers and supplements [10]. In recent years, intra-articular injections of MSCs [13], PRP [14], or the combination of MSCs and PRP [15] has been investigated for the treatment of OA and other bone injuries. Positive results demonstrate the safety and efficacy of PRP application in general surgeries, oral and maxillofacial surgeries, plastic surgeries and soft tissue healing in tendons, ligaments and muscles have been reported [2, 3, 16]. On the other hand, no beneficial efficacies of PRP treatment were reported in the healing of human Achilles tendinopathy [17], human Achilles tendons [18], or for canine bone formation [19]. Despite mentioned promising results, a consensus on the actual benefits of PRP has not yet been established. Such variation in outcomes related to PRP treatment could be attributed to some aspects of study design, such as sample sizes and control selections, in addition to the type of disease under investigation [1]. Another contributing factor could be the concentration and volume of PRP used during these treatments.

In this study, we have evaluated the effects of PRP concentration on the cell viability of the autologous canine bone-marrow derived multipotent mesenchymal stromal cells (K9BMMSCs) harvested from client-owned dogs with a history of OA in vitro. These findings will help streamline the methodology for using PRP as one of the standards of care treatment for injuries.

## Results

### Isolation and characterization of K9BMMSC cells

**Fig. 1:**
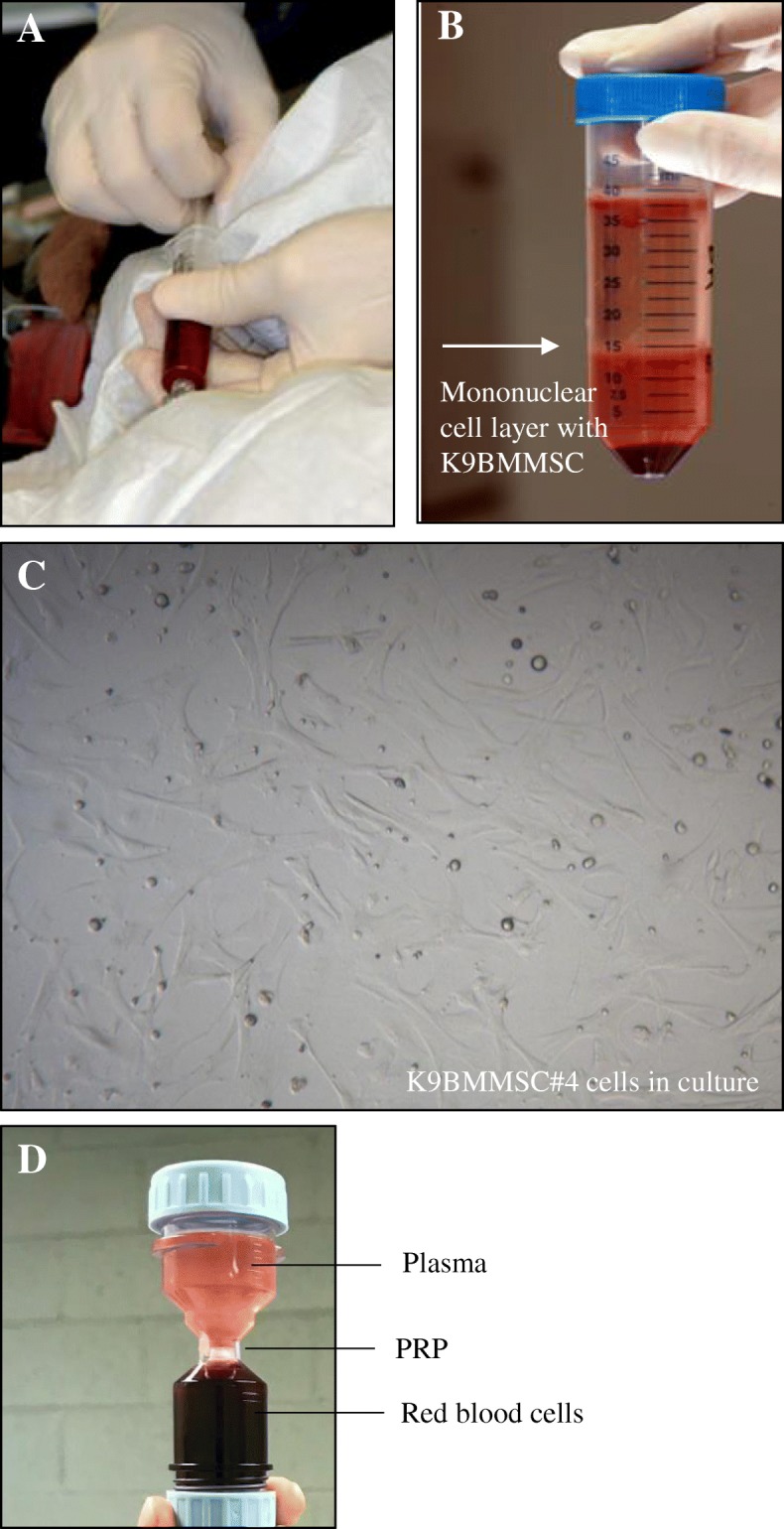
Isolation of MSCs from bone marrow and extraction of PRP from whole blood. **a** Bone marrow was harvested under sedation from the proximal humerus of client-owned dogs with osteoarthritis by a board–certified orthopedic veterinarian. **b** K9BMMSCs were isolated from the layer of cells (arrow) separated using LSM after centrifugation of bone marrow samples. **c** K9BMMSC#4 cells cultured in vitro in DMEM/F12 medium 7 days after seeding. **d** PRP was separated from whole blood using the Dr.PRP PRP kit. The separated layers of plasma (top chamber), PRP (center) and blood cells components (bottom chamber) in a Dr.PRP closed system tube after centrifugation. K9BMMSC: canine bone marrow-derived multipotent mesenchymal stromal cells; PRP: Platelet-rich plasma

**Fig. 2:**
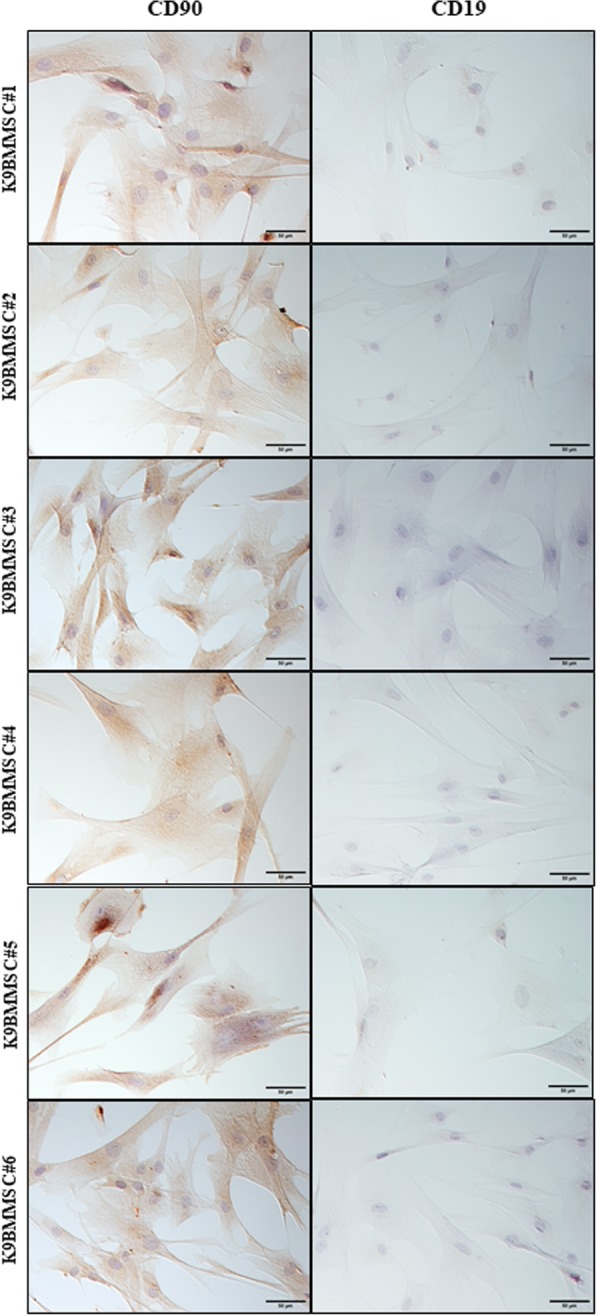
Characterization of K9BMMSCs by ICC. K9BMMSC cells were positive for CD90 (brown color, left panels) and negative for CD19 (right panel) expressions. Cells were counterstained with hematoxylin (blue color) for visualization of nuclei. Scale bar 50 μm. K9BMMSC: canine bone marrow-derived multipotent mesenchymal stromal cells

We successfully isolated K9BMMSC cells from six dogs diagnosed with OA (Table 1). K9BMMSC cells were isolated from the obtained bone marrow samples as shown in Fig. 1a. The mononuclear cell layer separated after centrifugation using LSM contained bone marrow-derived mononuclear cells (lymphocytes, monocytes, stem cells, progenitor cells, endothelial cells, and mesenchymal stromal cells) as shown in Fig. 1b. K9BMMSCs were cultured in complete DMEM/F12 media as shown in Fig. 1c and further characterized for expression of cell surface proteins using ICC staining. K9BMMSCs were confirmed to be positive for expression of CD90 (Fig. 2, left panel), a cell surface protein expressed in MSCs [20]. In addition, none of the K9BMMSCs expressed CD19 protein (Fig. 2, right panel), a transmembrane protein expressed only in B-lineage cells, which confirmed origin of isolated cells as MSCs [20].

**Table 1:** List of dogs enrolled in the study

Dog Number	Breed	Passage number of K9BMMSC cells used for the MTS experiments	PRP concentration (cells/ml)
#1	AM Staff	4	1.13 × 10^8^
#2	Bloodhound	4	4.5 × 10^8^
#3	Rottweiler	2	1.67 × 10^8^
#4	Labrador	4	Not available
#5	Mixed Breed	14	1.55 × 10^8^
#6	English Setter	3	5.29 × 10^8^

### Isolation and characterization of PRP

PRP was separated from blood collected from the same dogs as the K9BMMSC cells using the Dr.PRP PRP kit as shown in Table 1. Approximately 2–5 ml of PRP was separated from the central layer after centrifugation of blood (Fig. 1d). Platelets were counted using a hemocytometer, and the number of isolated platelets from each dog was reported in the Table 1, except PRP isolated from dog#4.

### Effects of PRP on cell viability and morphology of K9BMMSC cells

**Fig. 3:**
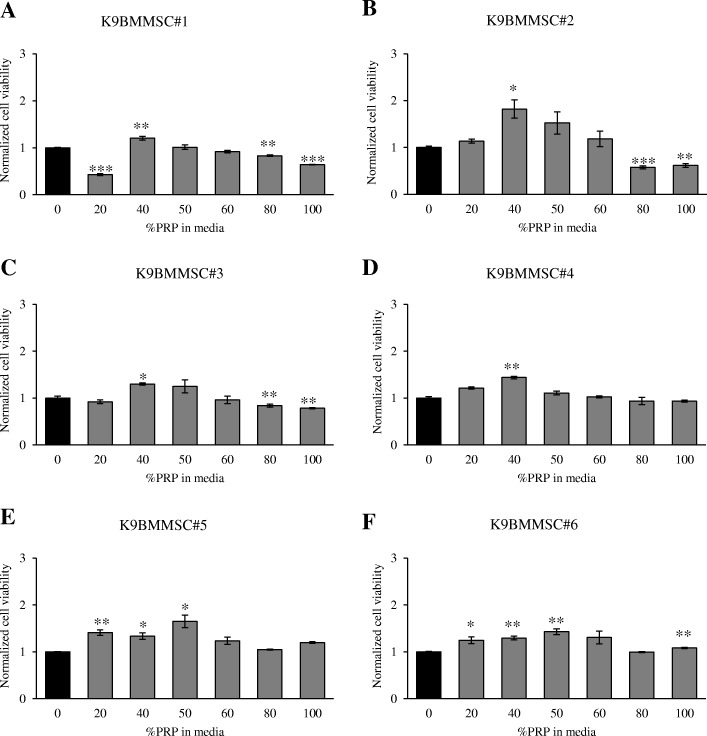
Effects of PRP on cell viability of K9BMMSC cells assessed by MTS assay. K9BMMSC cells were treated with increased concentration of PRP (0, 20, 40, 50, 60, 80, 100%) in DMEM/F12 complete media (v/v) for 72 h. Cell viability was determined by MTS assay. PRP at 40% concentration increased cell viability in all tested K9BMMSCs (**a**-**f**). Values represented here are mean ± S.E. of four replicates of PRP treated cells normalized to cells cultured in complete DMEM/F12 media only. Paired Student’s *t*-test was performed to compare differences in cell viability of PRP-treated K9BMMSCs to untreated cells. Significance was determined at ^*^p < 0.05, ^**^p < 0.01, and ^***^p < 0.001. K9BMMSC: canine bone marrow-derived multipotent mesenchymal stromal cells; PRP: platelet-rich plasma

The K9BMMSC cells were treated with PRP at concentrations of 20, 40, 50, 60, 80, and 100% (v/v) in complete DMEM/F12 media for 72 h and cell viabilities were assessed by MTS assay. PRP at 40% (v/v) concentration significantly increased cell viability by 20% (^**^p < 0.01) in K9BMMSC#1, 82% (^*^p < 0.05) in K9BMMSC#2, 30% (^*^p < 0.05) in K9BMMSC#3 cells, 43% (^**^p < 0.01) in K9BMMSC#4 cells, 33% (^*^p < 0.05) in K9BMMSC#5 cells, and 29% (^**^p < 0.01) in K9BMMSC#6 cells when compared to cells cultured in DMEM/F12 media only. In addition, PRP at 20 and 50% (v/v) concentrations also significantly increased cell viability by 40 and 64% (^**^p < 0.01, ^*^p < 0.05), respectively in K9BMMSC#5 cells and 24 and 42% (^*^p < 0.05, ^**^p < 0.01), respectively in K9BMMSC#6 cells. In K9BMMSC#1 cells, a significant 42% (^***^p < 0.001) reduction in cell viability was observed after treatment with 20% (v/v) PRP concentration. In remaining K9BMMSC cell lines, no significant differences between 20% or 50% (v/v) PRP concentration treated cells and untreated cells were observed. Likewise, 60% (v/v) PRP concentration did not have effect on cell viability of K9BMMSCs (Fig. 3). PRP concentrations at 80 and 100% (v/v) significantly suppressed viability by 17 and 36% (^**^p < 0.01, ^***^p < 0.001), respectively in K9BMMSC#1 cells, by 43 and 39% (^***^p < 0.001, ^**^p < 0.01), respectively in K9BMMSC#2 cells, and by 16 and 22% (^**^p < 0.01, ^**^p < 0.01), respectively of K9BMMSC#3 cells (Fig. 3a-c). Only 7% decrease in cell viability was observed in K9BMMSC#4 cells treated with 80 and 100% PRP concentrations, respectively, while an increase in cell viability by 4 and 19%, respectively, was observed in K9BMMSC#5 cells and 1 and 7% (^**^p < 0.01), respectively, was observed in K9BMMSC#6 cells.

**Fig. 4:**
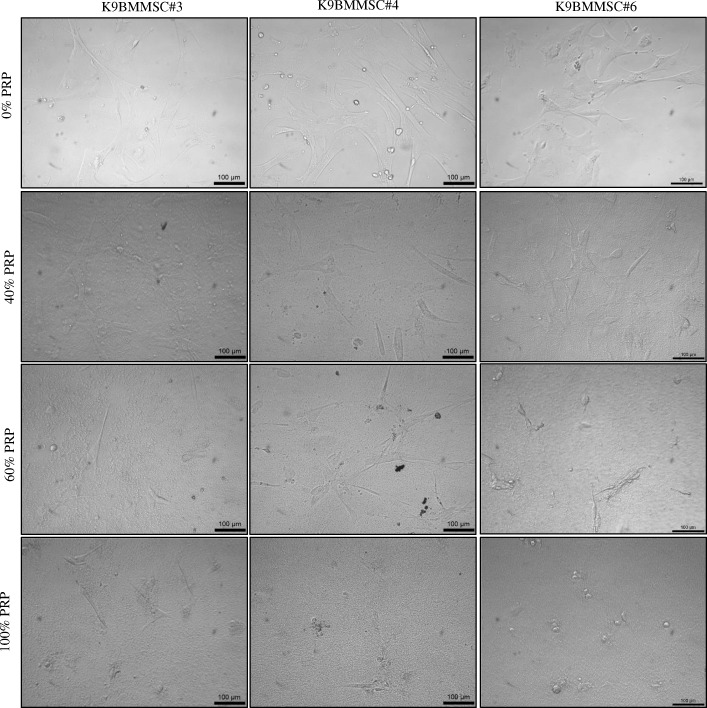
PRP affects morphology of K9BMMSC cells. Representative images of cell morphology of K9BMMSC#3, K9BMMSC#4, and K9BMMSC#6 cells cultured in 0, 40, 60, and 100% PRP for 72 h. Scale bar 100 μm. K9BMMSC: Canine bone marrow-derived multipotent mesenchymal stromal cells; PRP: Platelet-rich plasma

In addition, K9BMMSCs cultured in 0, 20, 40 and 60% PRP (v/v) concentration had elongated, spindle shaped morphology as shown in Fig. 4 and Additional file 1, while 80 and 100% PRP concentrations resulted in spherical morphology of the K9BMMSC cells as demonstrated in Fig. 4 and Additional file 1.

## Discussion

PRP is an attractive treatment option for patients with joint injuries, including tendon and ligament injuries, especially in the realm of sports medicine and orthopedic surgery [21]. Numerous clinical trials have confirmed the benefits of PRP and its products for the treatment of orthopedic diseases in companion animals as well as in humans [1, 22, 23]. PRP treatment alone [24], as well as in combination with MSC, has been demonstrated to promote bone regeneration [25]. Use of PRP and autologous cells are ideal for in vivo applications due to fewer immune compatibility, safety, and ethical concerns. PRP can be used as an alternate source of serum for culturing of MSC [26, 27]. A 5–10% platelet lysate in media was demonstrated to promote proliferation, expansion, colony formation, and differentiation of human MSCs compared to the fetal calf serum [26, 27].

Recent studies have focused on optimizing the concentrations of PRP used for treatments due to variable effects on viability of co-cultured cells in vitro [28–30] and in vivo [31]. PRP at 1–5% (v/v) concentration induced rapid proliferation of canine alveolar bone cells after 7 days in culture, while PRP at 30–100% (v/v) concentrations suppressed proliferation of alveolar bone cells [28]. Another study demonstrated that 2.5–20% PRP (v/v) concentrations stimulated proliferation and migration of primary rat Schwaan cells at day 3, 5 and 7 in vitro, while 40% PRP (v/v) concentration suppressed their proliferation and migration [29]. PRP obtained from normal healthy donors at lower concentrations (v/v) stimulated proliferation of primary oral fibroblasts and osteoblasts, while higher concentrations (v/v) of PRP were toxic to tested cells in vitro [30]. In addition to in vitro studies, in vivo studies have also confirmed the importance of using optimal PRP concentrations. PRP at low concentrations (2 × 10^6^ /mm^3^) promoted intestinal anastomotic healing in rats in vivo, while high PRP concentrations (5 × 10^6^ /mm^3^) impaired healing [31]. Similarly, positive effects on distal femur regeneration in New Zealand white rabbits in vivo was observed by optimal PRP concentrations (0.5–1.7 × 10^6^ /mm^3^); whereas low concentrations (0.16–0.37 × 10^6^ /mm^3^) was not effective and high PRP concentrations (1.85–3.2 × 10^6^ /mm^3^) inhibited bone regeneration [32]. Comparable to the previously published results in other cell lines and animal models, we observed that PRP concentrations with up to 50% (v/v) increased viability of K9BMMSCs cells, with PRP concentration of 40% (v/v) to be the most optimal. Our data demonstrated that viability of K9BMMSCs cultured at 60% (v/v) of PRP concentration was not different from cells cultured in complete DMEM/F12 media only. However, PRP at 80 and 100% (v/v) concentration were toxic to four of six tested cells. The exact mechanism of the effect of high concentration of PRP on cell viability is still unknown but is likely due to high concentrations of growth factors. It has been previously reported that PDGF and TGF-β1 growth factors concentrations are increased in concentrated PRP [31].

While our studies and numerous others demonstrate that concentrated PRP are not beneficial for cell culture, the described optimal concentrations vary between studies. These differences in the volume ratios, the quality and yield of PRP between studies can be attributed to preparation of PRP using variety of PRP preparation kits and procedures [33]. Furthermore, since PRP is isolated from different individuals, concentrations can be affected by difference in health status and condition of animal or person [34, 35]. Thus, it is required to further validate the optimal PRP concentrations to determine the optimal number of enriched platelets, the volume applied and its concentration of growth factors for clinical in vivo applications [36]. In conclusion, our observations indicate that PRP at 40% (v/v) concentration was beneficial for the cell viability of K9BMMSCs, but 80–100% concentrations (v/v) had opposite effects.

## Conclusion

Our results demonstrated that the concentration and volume of PRP affect the viability of K9BMMSC in vitro, which might have an effect on the treatment outcomes of OA in dogs in vivo. We confirmed that PRP at 40–50% concentrations (v/v) increased cell viability of K9BMMSCs, while high concentrations at 80–100% (v/v) inhibited cell viabilities in four out of the six tested K9BMMSC cell lines.

## Methods

### Animals

Six client-owned dogs with naturally-occurring OA were enrolled in this study according to the protocol approved by the Institutional Animal Care and Use Committee at the University of Tennessee (UT-IACUC). The owners signed the informed consent forms to agree to enroll their dog. Inclusion criteria at admission included clinical evidence of OA of the elbow or hip with a unilateral limb lameness greater than 5% between limbs on screening by force plate (FP) evaluation. Exclusion criteria included: presence of other major illness, orthopedic problems unrelated to our study, orthopedic surgery within the last 6 months, or skin infection of injection site for more than one week. Details regarding the dog’s breed, passage number of cells used in MTS assay and number of isolated platelets are presented in the Table 1.

### Bone marrow extraction

Approximately 4 ml of bone marrow was harvested from the proximal humerus of the lame limb of dog using a bone marrow aspirate needle (Fig. 1a) while the dog was under sedation. The procedures were performed by a board-certified veterinary specialist according to the approved UT-IACUC protocol. Bone marrow was mixed with 0.1% Citrate-dextrose solution (Santa Cruz Biotechnology, Dallas, TX) to prevent coagulation. The obtained bone marrow was immediately processed for isolation of K9BMMSCs.

### Isolation and expansion of K9BMMSCs

The harvested bone marrow mixture was diluted in 1x PBS and strained through a 70 μm nylon cell strainer (BD Falcon, Franklin Lakes, NJ) to remove blood clots. The mixture of bone marrow with PBS was slowly pipetted over 15 ml of Lymphocyte Separation Medium (LSM) (MP Biomedicals, LLC, Santa Ana, CA) and centrifuged at 1,000 rpm speed. The middle layer containing K9BMMSCs was gently isolated and washed with PBS (Fig. 1b). RBC lysis buffer was added to remove any residual red blood cells, followed by additional washes with PBS. The isolated K9BMMSCs were seeded in complete DMEM/F12 media (GE Healthcare Life Sciences, UK) supplemented with 10% fetal bovine serum, 100 IU/ml penicillin, and 100 μg/ml streptomycin and cultured in a 5% CO_2_ incubator at 37 °C. Cells were passaged when they reached 70–90% of confluence (Fig. 1c). Isolated K9BMMSC cells at passages 4–14 were used in our experiments.

### Extraction of PRP

PRP was isolated from whole blood using the Dr.PRP PRP kit (Dr.PRP USA, Missouri City, TX) following manufacturer’s instructions. Briefly, 18 ml of blood was drawn from the jugular vein with an anticoagulant syringe and placed in a blue PRP container. After centrifugation, separated PRP layer at the middle of the container (Fig. 1d) was extracted using a syringe. Isolated platelets were counted in a hemocytometer using the BMP solution following manufacturer’s instructions (LeukoChek, Gardner, MA). The isolated PRP was stored at − 80 °C freezer until further use.

### Immunocytochemistry (ICC)

K9BMMSC cells were cultured at a density of 5 × 10^5^**/**well on a 4-chamber slide and allowed to attach for 24 h. The ICC staining was performed according to a protocol as previously published [37]. Attached cells were washed in PBS and fixed in 4% paraformaldehyde solution for 10 min. Cells were rinsed twice in PBS and permeabilized using 0.1% Triton X-100 in PBS for 5 min. Cells were blocked in normal goat serum (Biogenex, Fremont, CA) and incubated with the anti-rat CD90 (AbD Serotec, UK) and anti-mouse CD19 (Millipore, Billerica, MA) primary antibodies overnight at 4 °C followed by incubation with secondary antibodies and then streptavidin conjugated with horseradish peroxidase (HRP, Biogenex, Fremont, CA) and visualized by a substrate 3,3`-diaminobenzidine (DAB, Vector Laboratories, Burlingame, CA). Cells were stained with diluted hematoxylin and following gradual dehydration were finally cover-slipped with xylene-based mounting media. Stained slides were evaluated, and images were captured by an Olympus DP73 camera (Hunt Optics and Imaging, Pittsburgh, PA) attached to a Leitz DMRB microscope (Leica) using cellSens Standard software (Olympus, Center Valley, PA).

### MTS assay

K9BMMSC cells were plated at a density of 5,000 cells/100 μL/well on a 96-well tissue culture plate in complete DMEM/F12 media and allowed to attach for 24 h. Cells were then treated with PRP at 0, 20, 40, 50, 60, 80, and 100% (v/v) concentrations in complete DMEM/F12 media and incubated for an additional 72 h. After treatment, cell viability of K9BMMSCs were measured using the MTS assay (MTS Cell Titer 96® Aqueous One Solution Cell Proliferation Assay, Promega Corporation, Fitchburg, WI, USA) following manufacturer’s instructions. The obtained absorbance at 490 nm (FLx800 plate reader, Bio-Tek Instruments, Winooski, VT, USA) from PRP-treated cells was normalized to untreated cells and reported as mean ± SEM.

### Image acquisition of cell morphology of K9BMMSCs

Images of K9BMMSCs in culture were captured by a MicroPublisher 3.3 camera (QImaging, Surrey, BC, Canada) attached to a Vista Vision microscope (VWR, Artisan Technology Group, Champaign, IL) using the Q-Capture Pro7 software (QImaging).

### Statistical analysis

Statistical analyses were conducted using the Student’s paired two-tailed *t*-test to establish difference between the PRP treated and control groups. Results were considered statistically significant at ^*^p < 0.05, ^**^p < 0.01, and ^***^p < 0.001.

## Additional file

Additional file 1: **Figure S1.** High PRP (v/v) concentrations inhibit K9BMMSCs in vitro. The K9BMMSCs treated with different concentrations of PRP (v/v) in DMEM/F12 complete media for 72 h. Images of the cell morphology changes in K9BMMSC#3, K9BMMSC#4, and K9BMMSC#6 cells cultured in 0, 20, 50, and 80% PRP (v/v). (PPTX 2630 kb).

## Data Availability

All data generated or analyzed during this study are included in this published article [and its supplementary information files]. Further information regarding the canine cell lines can be requested from the corresponding author.

## Abbreviations

BM: Bone marrow
DAB: 3,3`-diaminobenzidine
DMEM/F12: Dulbecco’s Modified Eagle Medium: Nutrient Mixture F-12
FGF: Fibroblast growth factor
HRP: Horseradish peroxidase
ICC: Immunocytochemistry
IGF-I, −II: Insulin-like growth factors I and II
K9BMMSC: Canine bone marrow-derived multipotent mesenchymal stromal cells
LSM: Lymphocyte separation medium
MSC: Mesenchymal stromal cells
MTS: Tetrazolium compound [3-(4,5-dimethylthiazol-2-yl)-5-(3-carboxymethoxyphenyl)-2-(4-sulfophenyl)-2H-tetrazolium, inner salt
OA: Osteoarthritis
PDGF: Platelet derived growth factor
PRP: Platelet rich plasma
TGF-β: Transforming growth factor-β
UT-IACUC: The Institutional Animal Care and Use Committee at the University of Tennessee
VEGF: Vascular endothelial growth factor
