# Supplementary figures and images for: Platelet-rich plasma affects the proliferation of canine bone marrow-derived mesenchymal stromal cells in vitro
Source: BMC Vet Res. 2019 Jul 30;15:269. doi: 10.1186/s12917-019-2010-x (PMC6668135; doi:10.1186/s12917-019-2010-x)

## Slide 1
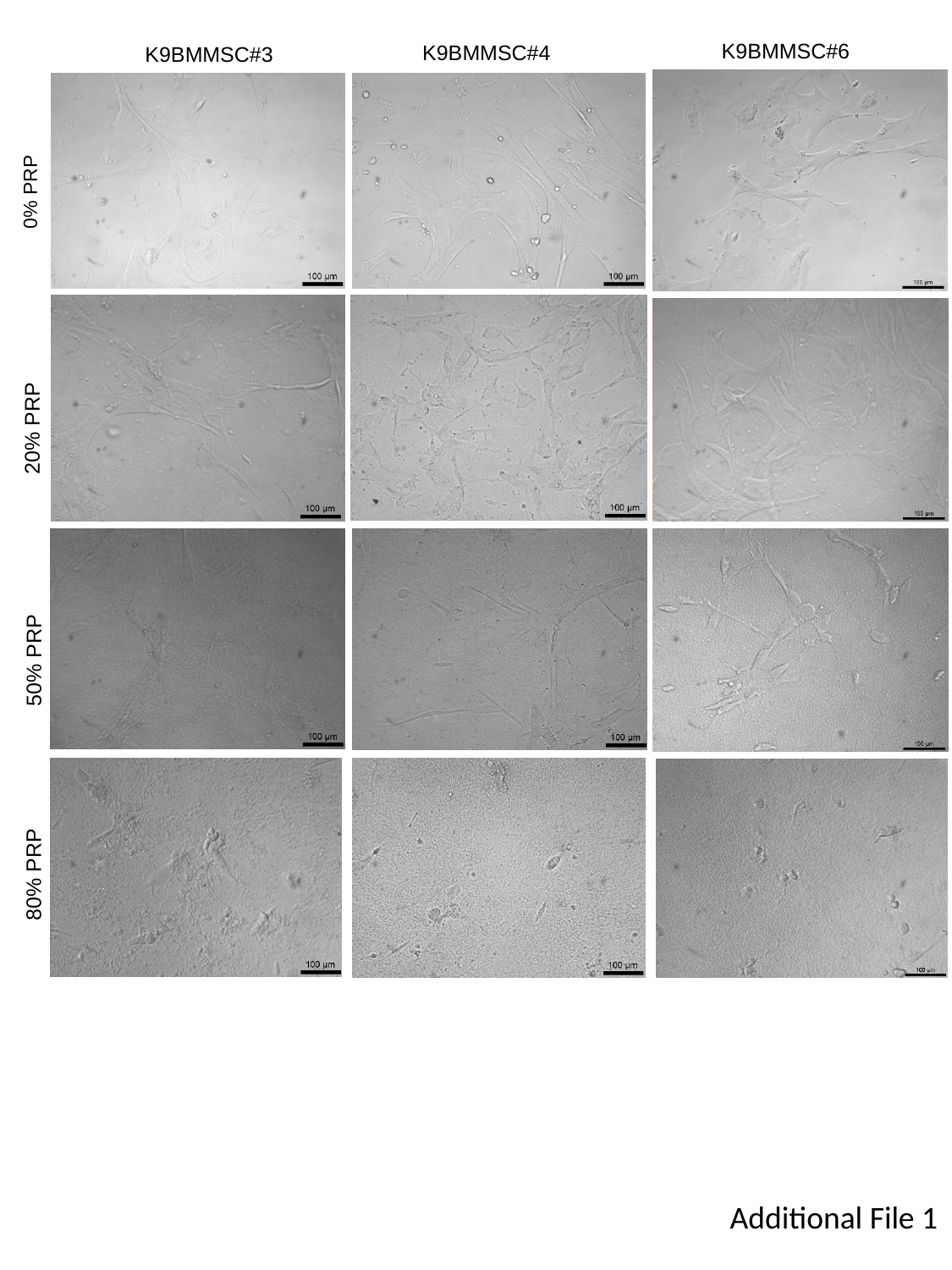

K9BMMSC#6
K9BMMSC#4
K9BMMSC#3
0% PRP
20% PRP
50% PRP
80% PRP
Additional File 1

Supplement: Supplementary file 1 — Figure S1. High PRP (v/v) concentrations inhibit K9BMMSCs in vitro. The K9BMMSCs treated with different concentrations of PRP (v/v) in DMEM/F12 complete media for 72 h. Images of the cell morphology changes in K9BMMSC#3, K9BMMSC#4, and K9BMMSC#6 cells cultured in 0, 20, 50, and 80% PRP (v/v). (PPTX 2630 kb). [file 12917_2019_2010_MOESM1_ESM.pptx]
